# Potential Probiotic *Escherichia coli* 16 Harboring the *Vitreoscilla* Hemoglobin Gene Improves Gastrointestinal Tract Colonization and Ameliorates Carbon Tetrachloride Induced Hepatotoxicity in Rats

**DOI:** 10.1155/2014/213574

**Published:** 2014-06-19

**Authors:** Prasant Kumar, Ayush V. Ranawade, Naresh G. Kumar

**Affiliations:** ^1^Molecular Microbial Biochemistry Laboratory, Department of Biochemistry, Faculty of Science, Maharaja Sayajirao University of Baroda, Vadodara Gujarat 390002, India; ^2^C.G. Bhakta Institute of Biotechnology, Faculty of Applied Science, UKA Tarsadia University, Bardoli, Surat Gujarat 394 350, India

## Abstract

The present study describes the beneficial effects of potential probiotic *E. coli* 16 (pUC8:16*gfp*) expressing *Vitreoscilla* hemoglobin (*vgb*) gene, associated with bacterial respiration under microaerobic condition, on gastrointestinal (GI) colonization and its antioxidant activity on carbon tetrachloride (CCl_4_) induced toxicity in Charles Foster rats. *In vitro*, catalase activity in *E. coli* 16 (pUC8:16*gfp*) was 1.8 times higher compared to *E. coli* 16 (pUC-*gfp*) control. *In vivo*, *E. coli* 16 (pUC8:16*gfp*) not only was recovered in the fecal matter after 70 days of oral administration but also retained antibacterial activities, whereas *E. coli* 16 (pUC-*gfp*) was not detected. Oral administration of 200 and 500 *μ*L/kg body weight of CCl_4_ to rats at weekly interval resulted in elevated serum glutamyl pyruvate transaminase (SGPT) and serum glutamyl oxalacetate transaminase (SGOT) levels compared to controls. Rats prefed with *E. coli* 16 (pUC8:16*gfp*) demonstrated near to normal levels for SGPT and SGOT, whereas the liver homogenate catalase activity was significantly increased compared to CCl_4_ treated rats. Thus, pUC8:16*gfp* plasmid encoding *vgb* improved the growth and GI tract colonization of *E. coli* 16. In addition, it also enhanced catalase activity in rats harboring *E. coli* 16 (pUC8:16*gfp*), thereby preventing the absorption of CCl_4_ to GI tract.

## 1. Introduction

Human gastrointestinal (GI) tract has a very complex microbiota, with approximately 500–1000 different species [1]. At birth, babies emerge from a sterile environment into one that is loaded with microbes as a result of which the infant's intestine rapidly becomes home to one of the densest populations of bacteria on earth [2]. The endogenous GI microbiota plays a fundamentally important role in health and disease, yet this ecosystem remains to be incompletely characterized [3]. The critical functions of the commensal microbiota include protection against irritable bowel syndrome, inflammatory bowel disease, colorectal cancer, and epithelial cell injury, regulation of host fat storage, and stimulation of intestinal angiogenesis [4, 5]. In GI tract, the microbial diversity changes from stomach to rectum. The microbiota of infants possesses three taxonomic groups, whereas healthy adults contain only five phyla and, amongst proteobacteria,* E. coli* is the predominant commensal microorganism present in the GI tract [2, 6–8].* E. coli* being a facultative anaerobe colonizes the GI tract at early stages and is proposed to facilitate the colonization of obligate anaerobes belonging to 22 different phyla by the creation of a reduced environment [9]. Many* E. coli* strains were demonstrated to have probiotic properties [10–13]. Previously, we isolated several* E. coli* strains from rat feces demonstrating characteristics such as acid tolerance, antibiotic susceptibility, nonpathogenicity, adherence capability, and antimicrobial activity to the Enterobacteriaceae family [13]. The* E. coli* 16 showed better adherence and acid tolerance capability along with other characteristics conferring this strain as potential probiotic [13].

Oxygen electron paramagnetic resonance (EPR) imaging technique showed that the GI tract environment fluctuates between anaerobic and microaerobic conditions [14]. In order to adapt the microaerobic environment of GI tract, bacteria downregulate or repress aerobic genes and simultaneously activate anaerobic genes [3].* E. coli* being a facultative anaerobe has aerobic respiratory control (ARC) system and fumarate nitrate reductase (FNR) system for aerobic and anaerobic conditions, respectively [15]. Aerobic bacterial respiration is essential for effective competition and colonization of* E. coli* in microaerobic environment of GI tract [16].

In oxygen poor habitats,* Vitreoscilla *sp., an obligate aerobe, survives due to efficient oxygen-binding kinetics of* Vitreoscilla* hemoglobin (VHb) encoded by* vgb* gene [17]. A heterologous expression of VHb in* E. coli* resulted in improved cell growth and protein production under microaerobic conditions [15]. Additionally, heterologous expression of* vgb* in* Enterobacter aerogenes* reduced H_2_O_2_ toxicity [18]. In* E. coli,* the protective role of VHb is mediated through oxidative stress regulator OxyR, which in turn activates VHb biosynthesis [19, 20]. VHb also has been shown to possess peroxidase activity [21, 22]. The chimera of superoxide dismutase and VHb protein rapidly detoxified reactive oxygen species (ROS) produced under oxidative stress conditions in* E. coli* [22].

Carbon tetrachloride (CCl_4_) causes tissue injury especially in hepatocytes by the formation of reactive trichloromethyl radicals [23]. Trichloromethyl radical reacts with molecular oxygen to form trichloromethylperoxyl radical and oxidizes lipid molecules by hydrogen abstraction especially in hepatocytes. The present study was designed to investigate the effects of* E. coli* 16 harboring* vgb* gene on GI tract colonization and CC1_4_ induced hepatotoxicity.

## 2. Materials and Methods

### 2.1. Bacterial Strains, Plasmids, and Culture Conditions

The bacterial strains and the plasmids used in this study are listed in Table 1.* E. coli* 16 isolate was maintained on Hichrome coliform agar and MacConkey agar plates (HiMedia, Mumbai, India).* E. coli* DH5*α* was used for constructing recombinant plasmids.* E. coli* BL21 was used for expressing the proteins. Luria-Bertani (LB) rich medium [5 g/L yeast extract (HiMedia, Mumbai, India), 10 g/L, Tryptone (HiMedia, Mumbai, India), and 10 g/L NaCl] and M9 minimal medium (12.8 g/L Na_2_HPO_4_ *·*7H_2_O, 3 g/L KH_2_PO_4_, 0.5 g/L NaCl, 1 g/L NH_4_Cl, 3 mg/L CaCl_2_, and 1 mM MgSO_4_) were used for plasmid construction and bacterial culture, respectively. NaNO_3_ (10 g/L) was added to the medium for induction of the* nar* promoter and 1 mM FeSO_4_ was added as a metal cofactor for VHb protein [18]. Plasmid-containing cells were grown in medium supplemented with 100 *μ*g/mL ampicillin.

### 2.2. Construction of Recombinant Plasmids and Transformation in* E. coli* 16

Green fluorescent protein (GFP) would be suitable as an* in vivo* marker for monitoring* E. coli* 16. Cloning of the* gfp* gene into the* Sma*I site of pUC8:16 results in* lacZ-gfp* fusion in which* gfp* is in frame with* lacZ* sequence. The recombinant plasmid was confirmed by restriction digestions. The plasmids pUC-*gfp* and pUC8:16*gfp* were independently transformed in the potential probiotic* E. coli* 16 using the CaCl_2_ method [24]. The transformants were screened by their fluorescence at 365 nm in ultraviolet transilluminator.

### 2.3. Preparation of* E. coli* 16 Culture and Cell-Free Extracts for Catalase Assays

Luria broth culture was grown as 60 mL of culture in a 100 mL flask. The cells were incubated at 37°C using agitation rates of 75 rpm and were treated with CCl_4_ (65 mM) at 0.4 to 0.5 O.D., that is, midlog phase, and incubated for 30 h. The cells were then harvested by centrifugation at 9,200 g for 2 min at 4°C. The cell pellet was washed once with 50 mM phosphate buffer (pH 7.0) followed by resuspension in the same buffer. The cells were subjected to sonication (Branson Sonifier Model 450) for a total period of 1 min at a pulse rate of 15 s in an ice bath, followed by centrifugation at 9,200 g at 4°C for 30 min to remove the cell debris. The supernatant thus obtained was used as cell-free extract for the catalase assay.

### 2.4. Catalase Assay of Cell-Free Extract

The cell-free extract was added in a cuvette followed by addition of 30 mM H_2_O_2_ prepared in 50 mM potassium phosphate buffer (pH 7.0). The decrease in absorbance was measured at 240 nm for 1 min to determine the catalase activity [25]. The molar extinction coefficient of 43.6 M/cm was used to determine the catalase activity and the activity was reported as units/min/mg of protein.

### 2.5. Animal Experiments

Male Charles Foster rats were housed in the departmental animal house facility under controlled room temperature (21 ± 2°C). The animals were fed chow diet and water* ad libitum.* The experiments were carried out after the approval of the Animal Ethical Committee of Department of Biochemistry, The MS University of Baroda, Vadodara (Approval no. 938/A/06/-CPCSEA). The guidelines of the Committee for the Purpose of Control and Supervision of Experiments on Animals (CPCSEA) were followed.

#### 2.5.1. GI Tract Colonization Experiments

Three-month aged rats were given drinking water containing streptomycin sulphate (5 g/liter) for 24 h to remove the existing resident facultative microflora and then starved for food and water for 18–20 h. The rats were divided into two groups and were fed approximately 10^9^ CFU of* E. coli* 16 (pUC-*gfp*) and* E. coli* 16 (pUC8:16*gfp*), respectively, in 1 mL of 20% sucrose once a day for up to 3 days. After the bacterial suspension was ingested, food and water were restored, and fecal plate counts were determined at regular intervals till the 70th day. Fecal samples were homogenized, serially diluted in 0.85% saline, and plated on Luria agar plates containing ampicillin (100 *μ*g/mL). After 24 h, the plates were inspected under UV light for the fluorescence. As soon as the reduction of fluorescent colonies of fecal samples was noted, rats were given ampicillin (50 mg/kg body weight) (days 23–25 and days 48–51) in drinking water [16, 26].

Selected fluorescent colonies from fecal samples were screened for* vgb* gene. The colony PCR was carried out on using a set of specific primers that anneal to a region of the* vgb* gene in* E. coli* 16 (pUC8:16*gfp*) and amplify a 714 bp fragment. Similarly, least dilution of fecal homogenate was plated on Luria agar plates containing lawn of* E. coli* DH5*α* to show the inherent antimicrobial property of* E. coli* 16 (pUC8:16*gfp*). The pUC8:16*gfp* is a nonmobilizable plasmid and does not get transferred horizontally [26].

#### 2.5.2. Effect of* E. coli* 16 (pUC8:16*gfp*) under Oxidative Stress

A total of 15 rats (14 to 16 months) were equally divided into five groups (*n* = 3). Group I served as normal control and was orally given saline for 3 days followed by 2 weeks interval up to 45 days. Group II served as potential probiotic* E. coli* 16 (pUC8:16*gfp*) where rats were fed the culture orally with saline for 3 days followed by 2 weeks interval up to 45 days. Group III served as normal control with CCl_4_ and was orally given saline for 3 days followed by 2 weeks interval up to 45 days. Further, two doses (200 *μ*L and 500 *μ*L) of CCl_4_ were given with olive oil as carriers at weekly interval and the antioxidant parameters in plasma and liver were monitored to assess the liver function. Group IV (potential probiotic* E. coli* 16 (pUC-*gfp*) with CCl_4_) served as vector control and the same procedure was followed as that of Group III. Group V [potential probiotic* E. coli* 16 (pUC8:16*gfp*)] with CCl_4_ served as test and the same procedure was followed as that of Group III. At the end of the 2nd dose, on the 3rd day, rats were mildly anesthetized and blood was collected via retroorbital sinus followed by plasma separation for further biochemical analysis. Later, animals were sacrificed by decapitation under mild anesthesia and liver was excised and stored at −80°C for further estimations.

#### 2.5.3. Assessment of Liver Function

Liver damage in the above mentioned groups of rats was assessed by estimating the plasma levels of serum glutamic oxaloacetic transaminase (SGOT) and serum glutamic pyruvic transaminase (SGPT) using commercially available mono single test IFCC, kinetic SGOT and SGPT test kits from Reckon Diagnostics Pvt. Ltd, Vadodara, Gujarat, India, as per the manufacturer's instructions.

#### 2.5.4. Estimation of Lipid Peroxidation and Catalase Activity

Liver samples (100 mg/mL) were homogenized in 50 mM potassium phosphate buffer and centrifuged at 10,000 rpm for 15 min. The supernatant thus obtained was used for estimating the lipid peroxidation levels and catalase activity. Catalase activity was expressed as enzyme activity per mg of protein. The protein concentration in each fraction was determined by modified Lowry method [27], using bovine serum albumin as standard. The mean malondialdehyde (MDA) content (*μ*mol/mg protein) was used as an indicator of lipid peroxidation and assayed in the form of thiobarbituric acid-reacting substances (TABRS) [28]. Catalase activity was measured using the method described previously [25].

#### 2.5.5. Microscopic Examination of Liver

Liver samples were fixed using 4% buffered paraformaldehyde followed by dehydration in graded alcohol series and embedded in paraffin wax. About 4-5 *μ*m thick sections were cut (by Leica RM 2155 Microtome) followed by staining with hematoxylin and eosin for examination using Leica microscope.

### 2.6. Statistical Analysis

Statistical evaluation of the data was performed by one way analysis of variance (ANOVA) followed by Bonferroni's corrections for multiple comparisons. The results were expressed as mean ± SEM using GraphPad Prism version 5.0 for Windows, GraphPad Software, San Diego, California, USA.

## 3. Results

### 3.1. Effect of* vgb* Gene Expression on* E. coli* 16 (pUC8:16*gfp*)

**Figure 1:**
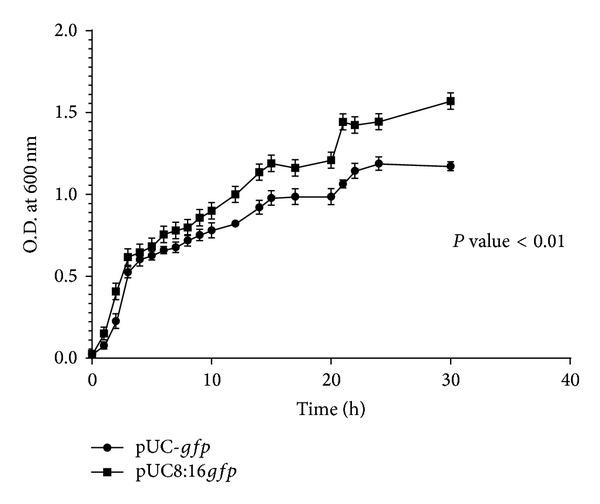
Growth curve of* E. coli* 16 pUC*gfp* and pUC8:16*gfp* transformants under static condition in flasks at 37°C. Microaerophilic condition was maintained by addition of NaNO_3 _(1%) in M_9_ medium.

A significant effect of* vgb* expression was observed as* E. coli* 16 (pUC8:16*gfp*) demonstrated an increase in growth rate under microaerobic condition compared to its pUC-*gfp* vector control (Figure 1).

### 3.2. *vgb* Gene Expression Enhances* In Vitro* Catalase Activity of* E. coli* 16 (pUC8:16*gfp*)

**Figure 2:**
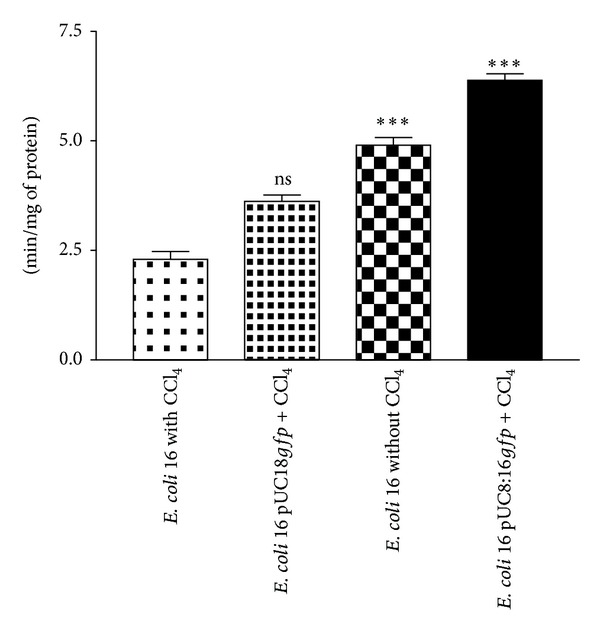
*In vitro* catalase activities of* E. coli* 16 containing pUC8:16*gfp* construct. Values are expressed as mean ± SEM (*n* = 4 each group) and analysis was performed using one way ANOVA. ***P < 0.001 and ^ns^ *P* > 0.05 compared to* E. coli* 16 with CCl_4 _treated groups.

In the presence of CCl_4_, catalase activity of* E. coli* 16 (pUC8:16*gfp*) was increased by 1.8-fold as compared to* E. coli* 16 (pUC8-*gfp*) vector control (Figure 2). This suggests that* vgb* gene was expressed under microaerobic environment and was functional in* E. coli* 16 (pUC8:16*gfp*).

### 3.3. GI Tract Colonization of* E. coli* 16 (pUC8:16*gfp*) in Rats Exposed to Intermittent Antibiotic Challenge

**Figure 3:**
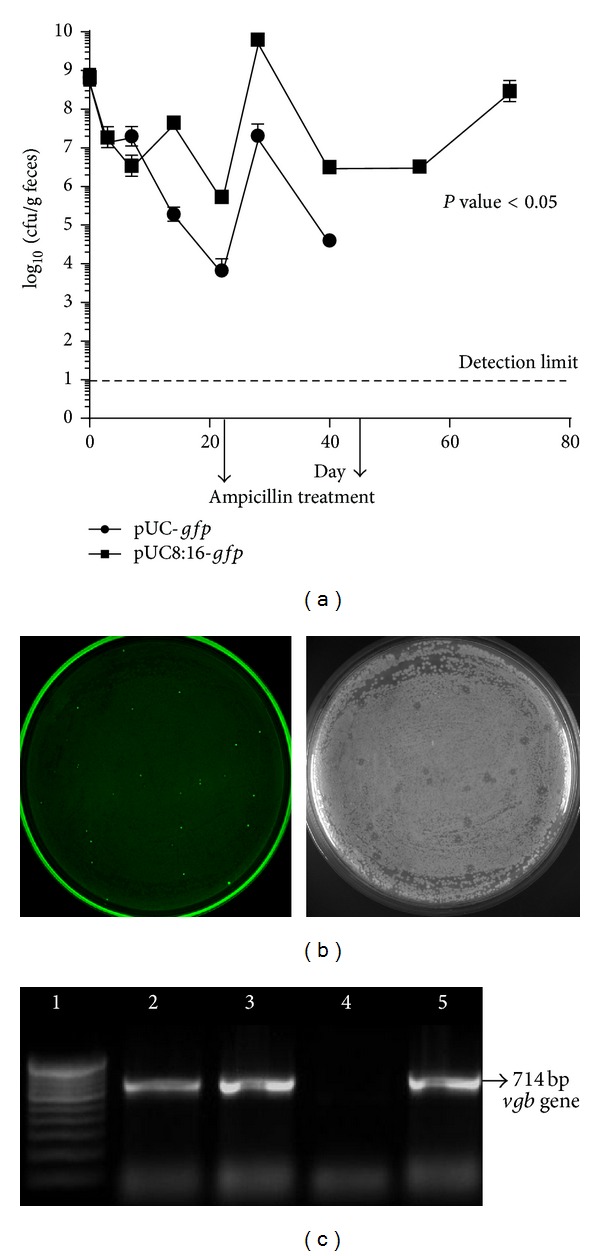
(a) Plate counts of fluorescent colonies in fecal samples of rats, following oral administration of* E. coli* 16 (pUC-*gfp*) and* E. coli* 16 (pUC8:16*gfp*) to respective groups of rats for the effects of antibiotic on the colonization process. (b)* E. coli* 16 strain transformed with pUC8:16*gfp* plasmid showed antimicrobial activity and expressed green fluorescent protein in fecal samples of Charles Foster rats. The plate (in the right side) showed antimicrobial activity under transmitted light and the same plate (in the left side) showed green fluorescent protein under ultraviolet light at 365 nm. The plate assay suggested that the transformants retained their antimicrobial activity even after passing through rat GI tract, and pUC8:16*gfp* plasmid did not affect the inherent antimicrobial activity of the* E. coli*. (c) Colony PCR for* vgb* gene in colonies showing fluorescence and antimicrobial activity in the plate. 1.5% agarose gel electrophoresis revealed 714 bp specific band of* vgb* gene (lanes: 2 and 3). Lane 1 represents 100 bp ladder; lane 4 represents plasmid pUC-*gfp* serving as negative control; lane 5 represents plasmid pUC8:16*gfp* serving as positive control for* vgb* gene.

*E. coli* 16 (pUC-*gfp*) transformants declined significantly in feces as compared to* E. coli* 16 (pUC8:16*gfp*) transformants. On the 21st day, the fecal* E. coli* 16 (pUC-*gfp*) counts were reduced by 100 times as compared to* E. coli* 16 (pUC8:16*gfp*) counts. After 22nd to 24th days, upon the first treatment of ampicillin, the counts of both* E coli* 16 (pUC-*gfp*) and* E. coli* 16 (pUC8:16*gfp*) transformants in feces were increased. However, after 48 days,* E. coli* 16 (pUC-*gfp*) was not detected, whereas* E. coli* 16 (pUC8:16*gfp*) counts remained constant. Moreover, after 51 days,* E. coli* 16 (pUC-*gfp*) was not detected even after ampicillin treatment whereas* E. coli* 16 (pUC8:16*gfp*) was detected even after second ampicillin treatment up to the 70th day (Figure 3(a)). Thus, the retention time of potential probiotic* E. coli* 16 (pUC8:16*gfp*) was significantly improved in the GI tract of rats. On the 48th day of postfeeding (Figures 3(b) and 3(c)), ampicillin resistant bacterial colonies were detected from fecal samples. These colonies demonstrated fluorescence as well as antimicrobial properties suggesting that both properties were retained in the* E. coli* 16 (pUC8:16*gfp*).

### 3.4. Effects of* E. coli* 16 (pUC8:16*gfp*) on Liver Function

#### 3.4.1. SGOT and SGPT Activity in Plasma

**Figure 4:**
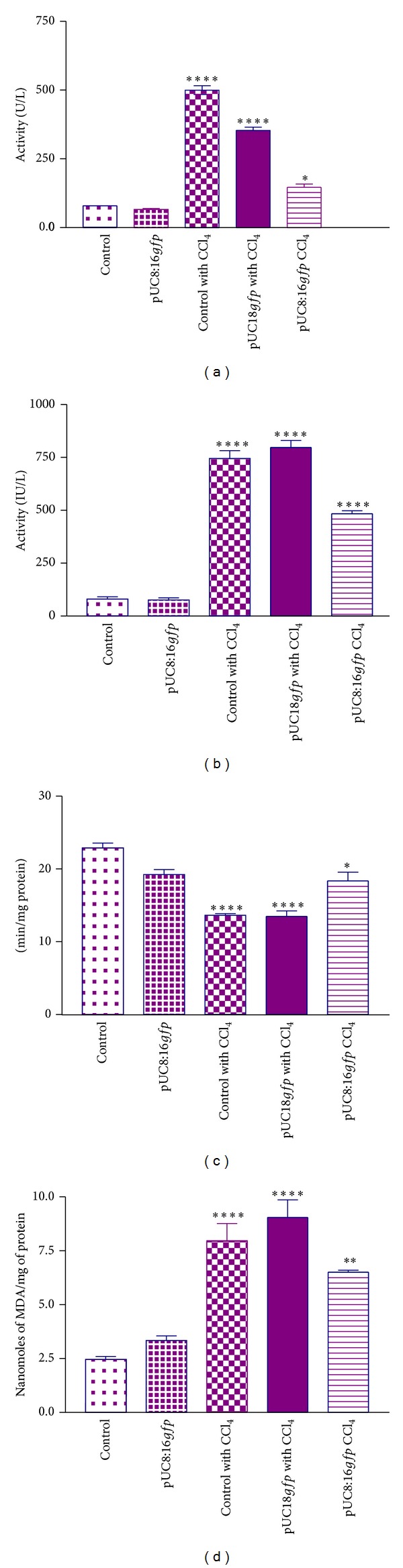
Effect of probiotic* E. coli *16 (pUC8:16*gfp*) encoding* vgb* gene on liver parameters: (a) serum glutamyl oxalacetate transaminase (SGOT) levels, (b) serum glutamyl pyruvate transaminase (SGPT) levels, (c) catalase activity, and (d) lipid peroxidation levels in different groups of rats treated with CCl_4_. SGOT and SGPT activities were measured in plasma, whereas catalase activity and lipid peroxidation levels were measured in liver homogenates. ****P < 0.0001, ***P < 0.001, **P < 0.01, and *P < 0.05 compared to Group I (control without CC1_4_ treatment). Values are expressed as mean ± SEM (*n* = 3 each group) and analysis was performed using one way ANOVA.

Oral administration of CCl_4_ to Groups III (normal control with CCl_4_) and IV (vector control) rats resulted in significantly elevated (*P* < 0.001) serum levels of SGPT and SGOT as compared to Group I (control) and Group II [prefed with potential probiotic* E. coli* 16 (pUC8:16*gfp*)] untreated rats. Treatment of CCl_4_ to Group V rats [prefed with potential probiotic* E. coli* 16 (pUC8:16*gfp*)] resulted in significantly decreased (*P* < 0.05) activities of SGOT and SGPT enzymes as compared to Group III and Group IV rats (Figures 4(a) and 4(b)). Rats treated with CCl_4_ and prefed with potential probiotic* E. coli* 16 (pUC8:16*gfp*) resulted in near to normal plasma SGPT and SGOT levels.

#### 3.4.2. Liver Lipid Peroxidation Level and Catalase Activity

Catalase activity was significantly decreased in the liver homogenate of CCl_4_ treated Group III and Group IV rats as compared to control groups. Potential probiotic* E. coli* 16 (pUC8:16*gfp*) Group V rats showed significantly (*P* < 0.05) increased catalase activity compared to CCl_4_ treated Group III rats (Figure 4(c)). Slight decrease in the mean MDA levels was found in the liver of Group V (CCl_4_-exposed) rats compared to Group III rats (Figure 4(d)).

#### 3.4.3. Histopathological Analysis of Liver

**Figure 5:**
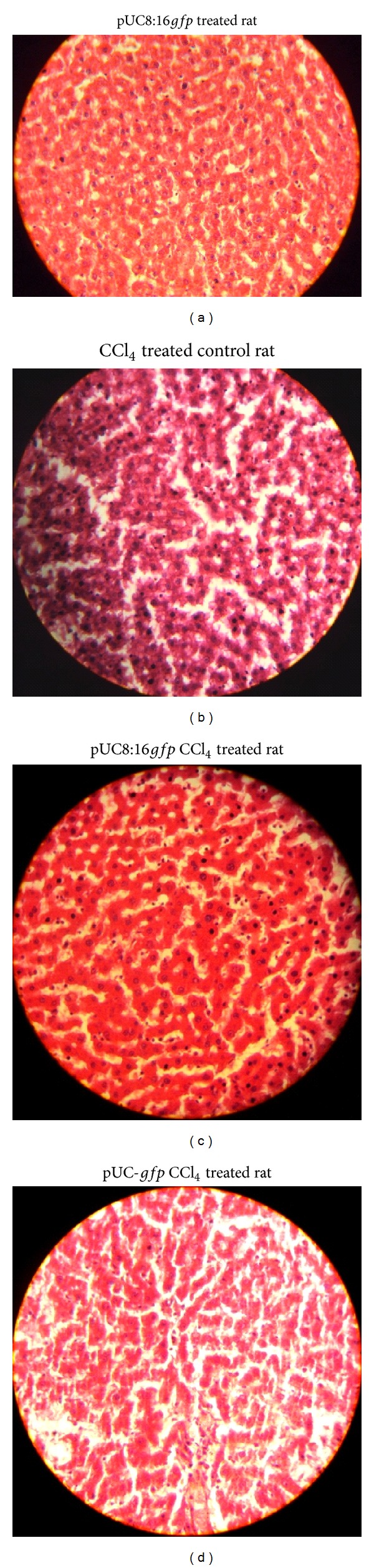
Effect of probiotic* E. coli* 16 (pUC8:16*gfp*) encoding* vgb* gene on CCL_4_-induced histopathological changes in different groups of rats: (a) photomicrograph of liver from Group II [control rat with probiotic* E. coli* 16 (pUC8:16*gfp*)], (b) photomicrograph of liver from Group III (rat treated with CCL_4_), (c) photomicrograph of liver from Group V [rat treated with CCL_4_ along with probiotic* E. coli* 16 (pUC8:16*gfp*)], and (d) photomicrograph of liver from Group IV [rat treated with CCL_4_ along with probiotic* E. coli* 16 (pUC-*gfp*)] as vector control. Haematoxylin and eosin were used for staining the paraffin-embedded sections.

Histopathological analysis of liver cells using hematoxylin and eosin stains in Group III and Group IV rats treated with CCl_4_ revealed extensive liver damage, characterized by the disruption of the lattice nature of the hepatocytes, damaged cell membranes, degenerated nuclei, disintegrated central veins, and damaged hepatic sinusoids as compared to the liver of Groups I and II (control) rats. However, in Group V rats [treated with CCl_4_ and prefed with potential probiotic* E. coli* 16 (pUC8:16*gfp*)], only minimal disruption of the hepatic cellular structure was observed (Figure 5).

## 4. Discussion

Probiotic bacteria exert their effects by competing with potentially pathogenic bacteria for ecological niches, thereby preventing their colonization. The exact mechanism of colonization of* E. coli* in the GI tract is not clear, but it is known that the respiration of* E. coli* in GI tract is very much essential for its successful colonization and competitiveness in the GI tract [16]. Colonization and competitiveness of facultative anaerobes, that is,* E. coli*, depend on their respiratory flexibility which in turn depends on high-affinity cytochrome bd oxidase. Previous study has shown that VHb improves the oxygen uptake rate of* E. coli* under microaerobic condition, by 5-fold and 1.5 increase of cytochrome bo_3_ and cytochrome bd oxidase, respectively [29]. In the study, VHb expression was enhanced by low oxygen tension via a fumarate and nitrate reductase regulator- (FNR-) dependent mechanism [29]. When a* vgb* gene is introduced into* E. coli*, the cell growth and yield of the target protein were increased significantly [16, 18, 30–34]. In the present study,* E. coli* 16 (pUC8:16*gfp*) plasmid expressing* vgb* gene was also found to increase the growth rate in microaerobic condition as well as improve the GI tract colonization and enhance the catalase activity. The expression of* vgb* gene significantly improved the colonization of potential probiotic* E. coli* 16 (pUC8:16*gfp*) in rat GI tract, possibly due to improved cell growth and better respiratory adaptation under low oxygen tension.

Superoxide radicals (O_2_^−^) formed within biological systems are toxic to living cells. Trichloroperoxyl radical (CC1_3_OO^•^) synthesized from (O_2_^−^) and CC1_4_ has a highly toxic effect on metabolic oxidizing activities presumably because of the electron-withdrawing nature of the trichloromethyl group [23, 35]. Heterologous expression of nonheme catalase in* Lactobacillus lactis* improved the antioxidant status and alleviated the risk of 1,2-dimethyl hydrazine induced colon cancer [36, 37]. Near to normal levels of SGPT and SGOT activity in CCl_4_ treated rats with* E. coli* 16 (pUC8:16*gfp*) plasmid demonstrate the protection of the toxic effects of CCl_4_ in the liver. Previous reports have suggested that the protective effects could be attributed to the peroxidase activity of VHb [21, 38]. Additionally, VHb is known to decrease the oxidative stress caused by H_2_O_2_ by enhancing the catalase activity [18]. The present study also found 1.8-fold increased activity of catalase contributed by* vgb* gene under* in vitro* condition. It has been shown that VHb in* E. coli* induces the expression of catalase-peroxidase G (*katG*) and superoxide dismutase A (*sodA*) genes, thereby protecting from damage caused by ROS [19]. In comparison, when* vgb* gene was expressed in* E. coli oxyR* mutant, the* vgb* expression was increased but the strain showed high sensitivity to oxidative stress without induction of antioxidant genes. Thus, oxidative stress regulator OxyR mediates the protective effect of* vgb* under oxidative stress [19].

## 5. Conclusion

The present investigation showed that* vgb* gene when expressed in a potential probiotic* E. coli* 16 strain increased its GI tract colonization, thereby improving its survival. In addition,* vgb* gene being an antioxidant, it detoxified the CCl_4_ in GI tract and thereby reduced the hepatotoxicity in rats. Hence, the retention of probiotics in GI tract is thus enhanced; it reduces the doses to maintain an effective probiotic count in the GI tract. These additional benefits may increase the efficiency of the probiotics making them more effective in minimum dose intervals.

## Figures and Tables

**Table 1:** Bacterial strains and plasmids.

Plasmids/strains	Relevant characteristics	Reference
Plasmids		
pUC-*gfp *	Derived from the high-copy number vector pUC18 by insertion of a modified *gfp* gene; Ap^r^	[26]
pUC8:16	Derived from the high-copy number vector pUC8 by insertion of a *vgb* gene; Ap^r^	[19]
pUC8:16-*gfp *	Derived from the high-copy number vector pUC8:16 by insertion of a *gfp* gene; Ap^r^	This study
Bacterial strains		
*E. coli* DH5*α*	F-endA1 glnV44 thi-1 recA1 relA1 gyrA96 deoR nupG Φ80dlacZΔM15Δ(lacZYA-argF)U169, hsdR17 (rK− mK+), *λ*−	[24]
*E. coli* BL21	*F* ′ *ompT hsdSB (rB− mB−) gal dcm *	[24]
*E. coli* 16	Wild type	[13]
*E. coli* 16 (pUC-*gfp*)	*E. coli *16 with pUC-*gfp* plasmid; Ap^r^	This study
*E. coli* 16 (pUC8:16-*gfp*)	*E. coli* 16 with pUC8:16-*gfp* plasmid; Ap^r^	This study

Ap^r^ represents ampicillin resistance; *gfp* represents green fluorescent protein; pUC8:16-*gfp* represents construct containing *vgb* gene tagged with *gfp.*
